# 

**Published:** 1915-06

**Authors:** 


### CONTENTS.

ORIGINAL COMMUNICATIONS—
Twilight Sleep, by W. F. Shallenberger, A. M., M. D., Atlanta, Ga. .......... 107
Two Uterine Stimulants, by Jams Robert McCord, M. D. .......... 116
The Alcoholic Psychoses, by James N. Brawner, M. D., Atlanta, Ga. .......... 118
Empyema of the Accessory Sinuses, by J. Calhoun McDougall, M. D., Atlanta, Ga. .......... 123
The Organization of National and Local Forces in the Campaign Against Cancer, by Curtis E. Lakeman, Executive Secretary, American Society for Control of Cancer .......... 126
Fulton County Medical Society, Regular Meeting June 3, 1915, R. R. Daly, M. D. .......... 133
Regular Meeting Fulton County Medical Society, June 17, 1915, by Allen H. Bunce, M. D. .......... 139
EDITORIAL .......... 143
NEWS AND NOTES .......... 146
SELECTIONS AND ABSTRACTS .......... 151
MEDICAL ITEMS .......... 162

